# Reshaping Smart Cities through NGSI-LD Enrichment

**DOI:** 10.3390/s24061858

**Published:** 2024-03-14

**Authors:** Víctor González, Laura Martín, Juan Ramón Santana, Pablo Sotres, Jorge Lanza, Luis Sánchez

**Affiliations:** Network Planning and Mobile Communications Laboratory, Universidad de Cantabria, 39005 Santander, Spain; lmartin@tlmat.unican.es (L.M.); jrsantana@tlmat.unican.es (J.R.S.); psotres@tlmat.unican.es (P.S.); jlanza@tlmat.unican.es (J.L.)

**Keywords:** data enrichment, linked data, data understandability, semantic annotation, data processing, smart cities

## Abstract

The vast amount of information stemming from the deployment of the Internet of Things and open data portals is poised to provide significant benefits for both the private and public sectors, such as the development of value-added services or an increase in the efficiency of public services. This is further enhanced due to the potential of semantic information models such as NGSI-LD, which enable the enrichment and linkage of semantic data, strengthened by the contextual information present by definition. In this scenario, advanced data processing techniques need to be defined and developed for the processing of harmonised datasets and data streams. Our work is based on a structured approach that leverages the principles of linked-data modelling and semantics, as well as a data enrichment toolchain framework developed around NGSI-LD. Within this framework, we reveal the potential for enrichment and linkage techniques to reshape how data are exploited in smart cities, with a particular focus on citizen-centred initiatives. Moreover, we showcase the effectiveness of these data processing techniques through specific examples of entity transformations. The findings, which focus on improving data comprehension and bolstering smart city advancements, set the stage for the future exploration and refinement of the symbiosis between semantic data and smart city ecosystems.

## 1. Introduction

In the current era of data-driven innovation, the increasing volume of information from heterogeneous sources, such as Internet of Things (IoT) deployments, open data portals, and social media platforms, provides a unique opportunity for data processing services to generate additional value [1]. At the same time, smart cities represent a pivotal evolution in urban life, harnessing advanced technologies to optimise infrastructure, services, and citizen experience [2]. High-value data has the potential to unlock new insights and increase efficiency at the core of this paradigm [3]. As cities become more interconnected, the integration of a wide variety of data enriched by semantic technologies such as the Next Generation Service Interfaces Linked Data (NGSI-LD) standard [4] becomes central to the success of smart cities. These high-value datasets and data streams provide both stakeholders and citizens with meaningful insights necessary to raise sustainability, enhance public services, and foster a more flexible and resilient urban framework [5]. By embracing the symbiotic relationship between smart cities and semantic data, urban habitats can transform into state-of-the-art, citizen-focused ecosystems, where decisions based on data push cities towards a future characterised by innovation, efficiency, and an improved quality of life [6].

In the midst of this data-rich landscape, however, challenges remain. Firstly, the sheer proliferation of heterogeneous data sources poses a challenge, as raw data often lack context and the absence of a standardised approach hinders their utility [7]. The value of data lies in their ability to create situational awareness that can be used to make optimised decisions, especially in the context of smart cities. There must be a proper framework that enables interoperability among the plethora of data sources for data processing techniques to have access to the full picture of a particular situation at a particular time (e.g., a true digital twin). Together, interoperability and dynamic discoverability facilitate competition and innovation, as users can effortlessly switch between data providers at their convenience [8]. Secondly, it is essential to apply semantic enrichment and linkage to supplement every data point with significant metadata concerning its quality, provenance, value, reputation, or limitations. Additionally, integrating knowledge with the raw information is crucial since the ultimate users of these data are the citizens and they need the data to be previously processed and served to them in the most natural and human-centric manner (“People want answers, not numbers”—Steven Glaser, UC Berkeley). Addressing these challenges is critical to realising the full potential of enriched data in the context of smart cities [9].

The focus of this article is twofold: firstly, to examine the complexities involved in utilising the data enrichment toolchain (DET) presented in [10] as a versatile framework to bridge the aforementioned gaps, and secondly, to analyse the implementation of several data processing modules and the resulting high-value data. The DET acts as a catalyst for implementing data enrichers and linkers tailored to meet the specific demands of smart cities. These modules are designed to use the full capabilities of NGSI-LD by harmonising diverse datasets and data streams and leveraging the semantic layer inherent to the standard. By basing our approach on linked data with a semantic context, we create a solid foundation that cultivates a mutually beneficial relationship between smart cities and enriched data. The modules, attuned to the demands of smart cities, play a pivotal role in making data more accessible and understandable. This interdependence between smart cities and data semantics not only addresses existing challenges but also paves the way for novel services and applications that resonate with the needs and aspirations of residents, by making data actionable beyond laboratories and, as a result, fostering citizen engagement.

All in all, the main innovations proposed in this paper have their foundations in the design and implementation of four data processing modules: The IoT data linker, that establishes relationships between NGSI-LD entities that originate from the same IoT device; the geolocation data linker, that links NGSI-LD entities to others located within a customisable distance; the reverse geocoding data enricher, that adds valuable metadata to NGSI-LD entities based on their *location* property; and the insight data enricher, that enhances the understandability and usability of NGSI-LD entities in accordance with well-known guidelines. Hence, the main contributions of this work in advancing the field of smart cities and data processing consist of making data more comprehensible and informative so that added-value services can be more easily deployed and tailored to the actual context and requirements of citizens. In this sense, the article is not only providing the general data enrichment framework but also specific examples that have been developed and validated through its integration within the DET and the processing of real smart city IoT data streams.

Returning to the aforementioned challenges that remain open, namely, interoperability, creation of situational awareness, and semantic enrichment, the overall solution described in this article directly addresses them. Firstly, the DET, which hosts the developed data processing modules, is leveraging the NGSI-LD standard not only to promote semantic interoperability through the use of a standard information model, but also to allow the exploitation of its linked-data principles to tackle the other two challenges. On top of this, the IoT data linker and the geolocation data linker are establishing relationships among data items, potentially coming from heterogeneous sources, so these data items are linked to their context (i.e., other data items). Thus, building situations out of independent but interrelated data. Conversely, the reverse geocoding data enricher and the insight data enricher are adding useful metadata and integrating external knowledge into the data items. Thus, increasing the amount of information available at each of them.

The remainder of the paper is structured as follows. Section 2 provides an overview of relevant literature on smart cities, data interoperability, and semantic enrichment. Section 3 presents the functional architecture of the DET, along with a specification of its core modules, with a strong emphasis on those developed within the scope of the article. Following this, Section 4 offers a detailed account of the implementation and deployment of these data enrichers and data linkers. The paper additionally outlines the resulting data obtained as the output of these modules. Lastly, Section 5 serves as the conclusion of the article.

## 2. Related Work

In this section, we provide a brief analysis of works and initiatives pertaining to the key concepts discussed in this article—smart cities, data interoperability enablers, and semantic enrichment.

### 2.1. Smart Cities

The concept of smart cities has been thoroughly explored and defined since seminal works such as [11,12]. The authors in [11] examine the integration of cutting-edge technologies to enhance different aspects of urban life. Similarly, Ref. [12] contributes to the discourse by greatly expanding the concept of smart cities, the services that can be offered, the enabling technologies and the implementation strategies. Broadly speaking, the concept of a smart city encompasses the strategic integration of information and communication technologies (ICT) to effectively manage and operate urban systems. Such technologies, like data analytics, IoT deployments, and interconnected networks, collectively enable cities to enhance sustainability, streamline governance, and improve the overall quality of life for their residents.

Comprehensive surveys, such as [2,13,14], have been instrumental in providing an overarching view of various smart cities around the globe, comparing their initiatives and highlighting common trends. Furthermore, the compelling case studies illustrated in [15,16,17,18] exemplify the implementation of smart city principles in cities worldwide. For instance, CitySense [15] merits recognition as a pioneering deployment from as far back as 2008, embedding devices in lamp posts in the city of Cambridge, MA. Later in 2010, the city of Oulu deployed a testbed consisting of outdoor sensor nodes [16]. However, a genuine deployment aimed at providing access to mobile nodes embedded in actual urban infrastructures, allowing for more realistic mobility experiments, did not occur until the SmartSantander project [18]. In recent years, other studies  [19,20,21,22,23] have demonstrated the potential of embedding technology and intelligent data-processing mechanisms for the improvement and optimisation of different aspects of urban management. These examples demonstrate the diverse and dynamic implementation of smart city concepts across differing geographical and socio-cultural contexts, contributing to the evolving narrative of urban intelligence and innovation.

Nevertheless, while the development of smart cities has undoubtedly demonstrated transformative potential, it is crucial to recognise and address inherent limitations that overlook different aspects related to the citizenry. As discussed in [24], certain developments in smart city initiatives have faced challenges in effectively translating technological advances into tangible benefits for all citizens. Using the definitions presented in [24], our article aims to focus the effort towards the notion of *smart citizens*. The lack of outreach to citizens is particularly pronounced for marginalised or less privileged groups, as highlighted by [25,26]. This critical perspective points out the possibility of inadvertently excluding a significant portion of the population when progressing the smart city paradigm, as access to the services offered is locked behind deep technological knowledge. Overcoming these challenges is crucial in establishing a genuinely citizen-centric smart city paradigm that enhances the well-being of all residents.

### 2.2. Interoperability Enablers

The concept of data interoperability has long been a driving force in the field of information systems. Already in 1995, the author of [27] recognised the need for it, understanding the potential for seamless data exchange between different systems. This early recognition formed the basis for further research towards achieving practical interoperability. Four years later, the authors of [28] further analysed the intricacies of data interoperability and its importance in promoting collaboration across different platforms. Although the perspectives were forward-thinking, practical executions were yet to materialise.

The turning point came in 2006 with the emergence of the concept of linked data and its four design principles [29], which brought a new approach to data interoperability. Linked data introduced the idea of connecting data elements in a web of relationships, prompting further research and development. As linked data gained popularity, the necessity for resilient ontologies became apparent. In more recent years, surveys like [7,30] have meticulously reviewed existing ontologies to gauge their suitability in developing interoperable applications, particularly in the context of the Internet of Things. The work carried out in [31] is a noteworthy effort towards advancing interoperability. It focuses on developing an ontology for OpenADR and specifically aims to enable interoperability in the domain of automated demand response, demonstrating the practical implementation of ontologies in achieving seamless data exchange. The evolution of interoperability, from visionary foresight to tangible implementations, illustrates its crucial role in modern data-driven ecosystems.

Numerous frameworks and enablers have been proposed to achieve data interoperability and address the complexities that arise in diverse data ecosystems. For instance, Ref. [32] presents a framework that introduces a systematic approach to data enrichment. On the other hand, the authors in [33] take an assertive stance by actively capturing and delivering information to facilitate dynamic interoperability with their big active data (BAD) system. In [34], the authors make a valuable contribution to the landscape through the introduction of a novel semantic virtualisation approach, which focuses on cross-domain IoT platform sensors and places emphasis on generating interoperable data acquisition plans. Ciavotta et al. [35] present a pipeline that prioritises performance-centric data enrichment and demonstrates its effectiveness in managing extensive datasets and large-scale studies.

Amidst these initiatives, the DET presented in [10] emerges as a complete and integrated solution. This work offers a set of tools that effectively enables information enrichment across various application domains. What sets the DET apart is its holistic approach, as it not only aims to provide a solid framework for heterogeneous data processing, but also offers a complete toolchain, from the data collection phase to the data enrichment phase, which seamlessly integrates into existing data ecosystems. This distinctive capacity situates the DET as a leading facilitator of interoperability, accommodating a wide range of data types and processing modules. Our work harnesses the strength of the DET, using it as the central framework for the implementation of specialised data enrichers and linkers. By adopting this framework, we rely on a solid, well-tested toolchain that comprises data from heterogeneous sources (IoT deployments, open data portals, social media) and is able to provide a standard interface with an NGSI-LD broker for both data consumption and reinjection. This decision enables our work to build upon the strengths of the DET, contributing to the advancement of interoperable solutions in the evolving landscape of data-driven innovation.

### 2.3. Semantic Enrichers

Semantic enrichers, linkers, and annotators are essential components for enhancing the depth and context of information within datasets or data streams, facilitating a more nuanced comprehension. This process involves augmenting data with semantic information, providing additional layers of meaning and relationships with other data. Different authors have explored various paths within semantic enrichment, each tailored to certain domains and applications.

Gutierrez et al. [36] make a contribution to the field by presenting a textual data enrichment framework aimed at enabling recommender systems. Specifically designed for this application, the approach uses natural language processing (NLP) techniques to validate and enrich textual data, thereby improving the capabilities of recommendation algorithms. In the domain of historic buildings, Ref. [37] concentrates on showcasing how semantic enrichment can effectively manage and enhance data related to architectural heritage, especially 3D models of the buildings. In [38], the authors bring data enrichment to the field of genetics, and introduce the Semantic Annotation Platform with Provenance (SAPP), which leverages linked data to process and analyse genome data. Mylonas et al. [39] present a pilot project focused on the domain of viticulture, which utilises automatic metadata enrichment to improve services and information pertinent not only to the smart agriculture domain, but more specifically to the wine industry. On the other hand, Ref. [40] explores the semantic enrichment of structured data, with an emphasis on utilising external semantic knowledge sources. The application of their approach holds great potential in diverse artificial intelligence (AI) domains, which highlights how semantic enrichment can augment and enrich data for various applications. Finally, Ref. [41] introduces a platform for developing smart applications based on diverse data sources to support a semantically enriched data model for effective data analysis and integration. This platform also includes a semantic layer that takes the gathered data and represents it utilising semantic web technologies for the annotation and linking of data, and to facilitate more accurate and meaningful analysis. Indeed, they develop some applications focused on improving urban air quality monitoring by utilising IoT data and applying semantic enrichment techniques, which have similar behaviour to the data enrichment modules described in this article.

Through a range of unique perspectives and methodologies, each of these works showcases the versatility and applicability of semantic enrichment in different domains and contexts. However, there are two important shortcomings that we address in our work. Firstly, while they are using proprietary semantic data models, thus creating proprietary knowledge graphs, we have leveraged standard information modelling that improves the interoperability of the resulting architecture. Secondly, they restrict the data semantic enrichment to the transformation of raw data into a semantic format (RDF-based modelling), while we also leverage the intrinsic extensibility of semantic data, through the adoption of linked data principles, to annotate the results of data processing into the available data, so that consumers can benefit from that processing without having to perform it themselves.

In this work, we explore several key domains that align with the challenges and opportunities of the contemporary era of data-driven innovation. In addressing the intricacies of smart cities, our linkers and enrichers stand as practical solutions rooted in the challenges and limitations discussed in [24,25]. Within the realm of interoperability, we chose to found our work in the framework provided by [10]. Our commitment to utilising the complete range of interoperability capabilities motivated this decision, guaranteeing that our linkers and enrichers function within a standardised and adaptable framework able to homogenise data from domains of diverse nature. Entering the realm of semantic enrichment, our approach goes beyond domain-specific boundaries. While drawing insights from domain-specific data enrichers [36,37,38,39,40], our work spans diverse applications and areas of knowledge. Our linkers and enrichers act as catalysts, transforming NGSI-LD data into actionable intelligence and enhancing the symbiotic relationship between smart cities and semantic data.

## 3. Architecture

In this section, the functional architecture of the DET is described, along with a thorough explanation of its role as the key enabler for data enrichment and linking. Moreover, it also expands on these two concepts and provides a high-level view of the modules developed within the scope of this article.

### 3.1. Data Enrichment Toolchain

The main objective of the DET is to enable the enhancement of datasets and data streams by way of enrichment mechanisms based on the application of linked data, semantics, and AI technologies.

**Figure 1:**
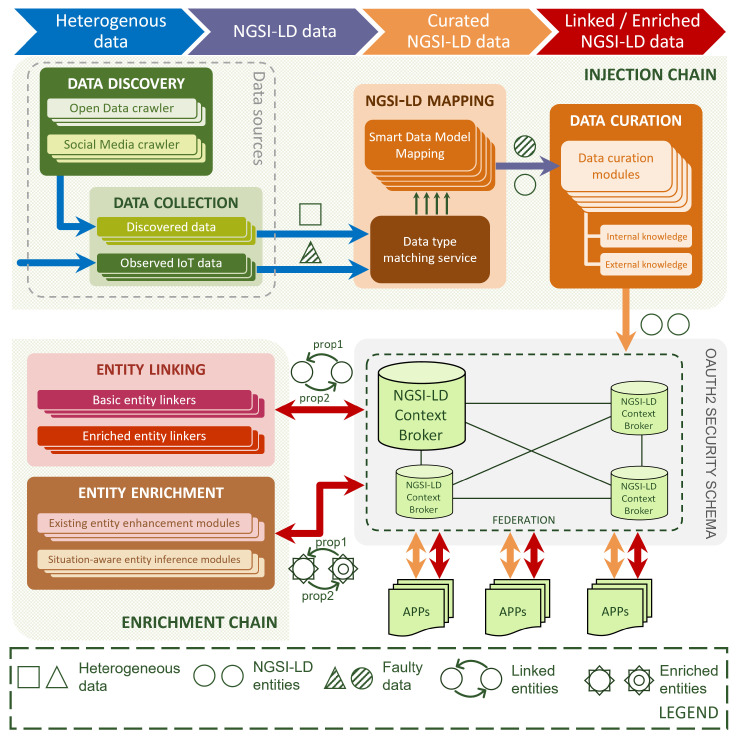
Functional architecture of the data enrichment toolchain (source: [10]).

Figure 1 depicts the DET functional architecture and illustrates the flow of data through different modules. The DET is composed of microservices that progressively transform and enhance the data. Particularly, the aim of the data enrichment phase, which is the focus of this paper, is to improve the quality and value of the original information. In general terms, the DET can be seen as a pipeline with a set of modules that each target an atomic step within the overall process.

The core components of the architecture, as seen in Figure 1, are the injection chain, the context broker (one or multiple in a federation), and the enrichment chain. The injection chain is responsible for transforming raw data into curated NGSI-LD data. The processed data can be accessed by external applications through the context broker, which facilitates communication, storage, and historic data management. Finally, the enrichment chain handles the linking and enrichment of NGSI-LD data obtained through the broker. A more detailed explanation of each step is provided below:

- **Data discovery and collection** modules acquire raw data from heterogeneous sources. These may include, but are not limited to, IoT-based deployments, social media, web-stored data, statistical catalogues, or meteorological agencies. The output of this phase consists of the raw data collected from various data sources, which are, by definition, heterogeneous in both type and format.
- **NGSI-LD mapping** modules transform the raw data into the NGSI-LD information model, and more specifically, the resulting data are compliant with FIWARE’s smart data models initiative [42]. The transformed data are then forwarded to the next phase.
- **Data curation** modules ensure that the data injected in the NGSI-LD context broker is adequate to be processed by data processing modules. As an example, curation may include data quality mechanisms such as outlier detection, deduplication, loss management, or taggers for data quality metrics (accuracy, timeliness, and so on). These modules inject their resulting clean data into the NGSI-LD context broker.
- **Entity linking** modules create NGSI-LD relationships between two or more NGSI-LD entities, regardless of their data source. This is achieved by finding and establishing common aspects among data, whether semantic, spatial, temporal, or otherwise. These relationships facilitate any further processing by simplifying navigation through connected data. Once the linking process is finished, the newly linked data are injected back into the NGSI-LD context broker.
- **Entity enrichment** modules generate new NGSI-LD entities or new NGSI-LD properties in existing entities. This is usually achieved by leveraging information from external knowledge sources. These modules are typically specific to a particular domain and are designed with a specific application or use case in mind; however, this is not always the case, as domain-agnostic enrichment is also a possibility. After the enrichment process is complete, the enhanced data are injected back into the NGSI-LD context broker.

The DET is further enhanced by several security mechanisms. The NGSI-LD context broker is secured behind an OAUTH2-based schema, which enables authorisation restrictions based on JSON Web Tokens (JWTs), and the communication is encrypted with Transport Layer Security (TLS).

The DET used in this article is publicly available and can be found at [43].

### 3.2. Data Linking and Enrichment

As previously mentioned in the functional architecture of the DET, the injection chain is aimed at harvesting, homogenising, and curating diverse data from heterogeneous sources, but it does not provide a clear value-added service other than potentially republishing the data in a more structured manner. In a similar fashion, the NGSI-LD context broker stores the data and manages access to the datasets and data streams, but no data augmentation service is provided at that stage. The true value of the DET is revealed during the enrichment chain phase, where the enrichment and linking modules represent the culmination of the entire process by enhancing the value of the data based on their individual focus. It is only at this point that applications accessing the augmented data are able to reap the benefits of using the DET as their source.

Data linkers and enrichers, on the other hand, also rely on the services provided by the rest of the DET modules in order to function properly. The most straightforward example is the interaction with the NGSI-LD context broker; the data processing modules obtain the data to be enhanced from an NGSI-LD interface, which has to be provided by one such broker. Not only that, but the output of the modules (i.e., NGSI-LD enhanced entities) also needs to be stored after the enhancement process, which is again a service provided by the NGSI-LD context broker. Additionally, enrichment and linking modules are usually tailored to a specific data type, or at least a data format. The enhancement process would be a significantly more costly task were it not for the prior transformation and curation provided by the injection chain. Working with well-known NGSI-LD data models allows the data enhancement modules to focus on their specific tasks, avoiding the waste of computational resources (and human effort) on tasks better suited for the modules comprising the injection chain phase of the DET.

Overall, we believe that using the DET as an enabler for our modules is a sensible choice. This will simultaneously increase the overall value of the toolchain and allow us to benefit from the services provided by the previous steps. The following subsections contain a high-level description of the data linkers and enrichers developed within the scope of this work. These will later be instantiated as DET modules, as discussed in Section 4.

#### 3.2.1. IoT Data Linker

**Figure 2:**
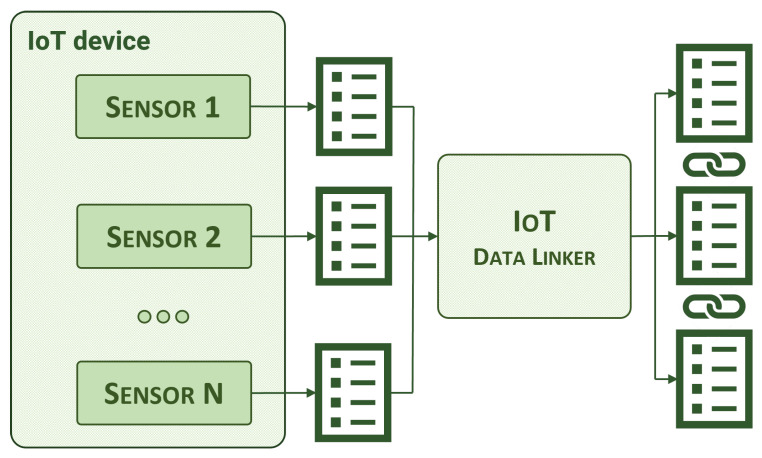
IoT data linker, high-level view.

The IoT data linker establishes relationships between entities that share the same device of origin. In IoT infrastructures, sensors and devices often generate different types of measurements. For instance, it is not uncommon to see a device that provides both temperature and battery status measurements. However, by default, these two entities are not semantically linked in any way. The aim of the IoT data linker is to identify these situations, as illustrated in Figure 2, and connect entities through an NGSI-LD relationship.

The IoT data linker directly operates on data extracted from an NGSI-LD context broker. It can retrieve batches of data by making queries through the standard interface (a HyperText Transfer Protocol (HTTP) GET request to the /entities endpoint) or subscribe to the types of data it is interested in (through an HTTP POST request to the /subscriptions endpoint). NGSI-LD entities are processed individually, and the IoT data linker can identify entities from the same physical device thanks to the ID scheme used in the DET. The entity ID is structured to allow for query filters using regular expressions, and the response will include entities whose ID matches the query. In the case of this component, we can extract entities with the same device of origin, provided that the original collected entities include an identification for this device. After building a list of related entities, we insert them into an NGSI-LD relationship, creating the semantic link. Finally, the linked NGSI-LD entity is reinjected into the NGSI-LD context broker by sending an HTTP POST request to the /entities endpoint.

#### 3.2.2. Geolocation Data Linker

**Figure 3:**
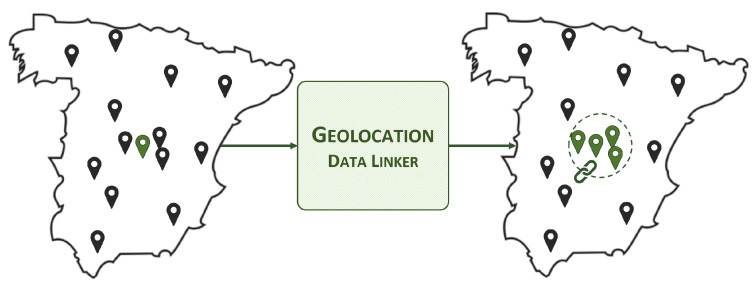
Geolocation data linker, high-level view.

The geolocation data linker links NGSI-LD entities to others located within a certain distance. Typically, there is no simple way to determine which other entities are in proximity to a given entity. The only way to achieve this is by manually checking the coordinates, preparing the range or area, and making a series of specific requests to the NGSI-LD context broker. The goal of the geolocation data linker is to simplify the process by automating necessary requests and linking entities through an NGSI-LD relationship in advance, as shown in Figure 3. This benefits various actors, including applications, linkers, enrichers, and even users skimming the data.

The geolocation data linker extracts NGSI-LD entities directly from the broker by combining queries and subscriptions, much like the IoT data linker. Each NGSI-LD entity is processed individually. The module reads the location value of the entity and performs a geolocation-based query to the broker, leveraging NGSI-LD geographical query capabilities. The entity’s location serves as the centre of a circle, with the radius determined by a configurable parameter (called *distance*) that can be easily adjusted. The broker responds with a list of entities found within the specified range of the original location. This list is then inserted into an NGSI-LD relationship within the original entity, completing the linking process. The NGSI-LD entity is then reintroduced into the NGSI-LD context broker by making an HTTP POST request to the /entities endpoint.

#### 3.2.3. Reverse Geocoding Data Enricher

**Figure 4:**
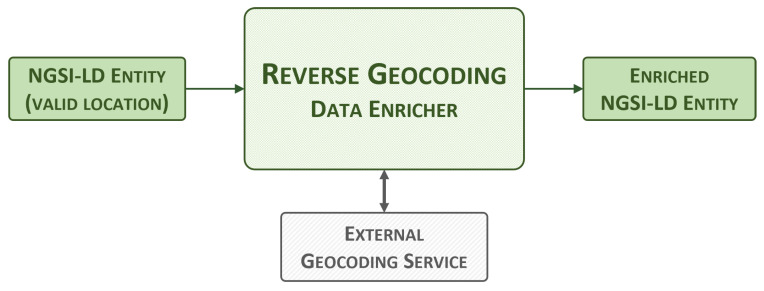
Reverse geocoding data enricher, high-level view.

The reverse geocoding data enricher, shown in Figure 4, can enrich any NGSI-LD entity that includes a *location* property. This property is widely used and is included by default in all smart data models as it allows for the exact coordinates of an object or event represented by an entity to be pinpointed. This data enricher uses the *location* property to obtain precise information about the position of an entity. This information may include the country, region, city, postal code, and/or street number, if applicable. The process of obtaining this knowledge from a set of coordinates is known as reverse geocoding.

To obtain these values, the reverse geocoding data enricher uses the third-party open-source service Nominatim [44], which relies on OpenStreetMap data. This module extracts NGSI-LD entities directly from the NGSI-LD context broker by combining queries and subscriptions. It reads the location value of the entity and performs a reverse geocoding request to the Nominatim application programming interface (API). This request is highly flexible, as it is able to translate all GeoJSON geometries (i.e., Point, LineString, Polygon, and so on). The acquired information is then used to update the *address* and *areaServed* properties with specific country, city, state, postal code and road values, which are commonly found in smart data models. The enriched entity, now including the new properties, is then sent back to the broker via an HTTP POST request to the /entityOperations/upsert endpoint.

#### 3.2.4. Insight Data Enricher

**Figure 5:**
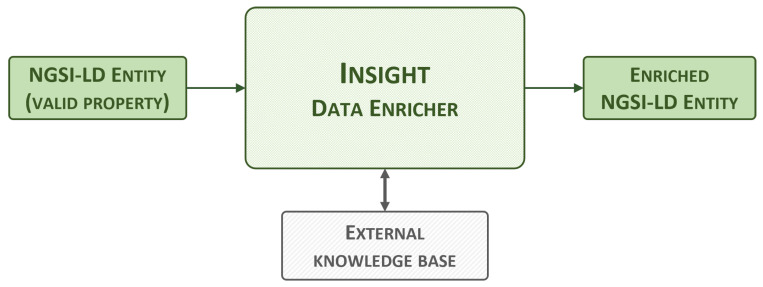
Insight data enricher, high-level view.

The insight data enricher, whose high-level view is shown in Figure 5, enriches specific NGSI-LD entities based on their corresponding smart data model. Some models have properties that include a numerical value and, if necessary, metadata such as a unit or timestamp. Occasionally, these numerical values have a non-obvious meaning, making them difficult for humans to understand. For example, an air quality measurement may display an NO2 value of 67.7 with a unit code of GP. There is a high chance of this not being understood by the average citizen.

This component adds new metadata to these types of properties to simplify the user experience. This is achieved by implementing a sub-property with a string type that provides a brief description of the enriched value. In the example given, the string “dangerous” is added to the value of 67.7 to explain in simple terms that it is not a safe level of NO2. The thresholds that separate two adjacent levels (e.g., safe versus unsafe) have been selected based on established and reputable sources. For instance, air quality thresholds are based on guidelines published by the World Health Organization (e.g., [45]). These sources are also included as metadata for traceability.

The NGSI-LD entities are extracted directly from the NGSI-LD context broker, combining queries and subscriptions. Each NGSI-LD entity and eligible property is processed individually. The value of the property is compared to pre-defined thresholds, and a new sub-property is generated and added to the original value. The enriched entity, now including one or more new sub-properties, is then reinjected into the broker via an HTTP POST request to the /entityOperations/upsert endpoint. The insight data enricher currently supports the *AirQualityObserved*, *SoundPressureLevel*, *Temperature*, and *TrafficFlowObserved* smart data models. Within air quality entities, *PM2.5*, *PM10*, *O3*, *NO2*, *SO2*, and *CO* are supported. Similarly, in temperature entities, both Celsius and Fahrenheit are supported.

## 4. Implementation and Validation

This section discusses the implementation and deployment of the modules described in Section 3, diving into the finer details of the setup employed in the realisation of this work. Furthermore, it provides a precise description and discussion of the experimental results derived from the usage of these modules.

### 4.1. Experimental Setup

The setup envisioned for the implementation phase comprises three core elements, all of which have been identified and described in Section 3. The deployment details of these elements are explained below:

1. **DET**: This element is the key enabler of the entire process, as it collects raw data from a smart city domain, maps them into NGSI-LD fit for the subsequent modules, and provides a layer of data curation. The DET has been downloaded from [43] and deployed in a Docker container within an Ubuntu 20.04.5 LTS machine (2 CPU cores, 2.40 GHz clock, 16 GB RAM).
2. **NGSI-LD broker**: The context broker provides both an NGSI-LD interface and storage capabilities, including historic data management. We have selected the Scorpio Broker [46] as it provides all the capabilities we require. The DET, in its injection chain phase, stores the curated NGSI-LD entities in the broker, which sends a notification to the processing modules due to NGSI-LD’s subscription/notification feature. The Scorpio Broker is also deployed in a Docker container within an Ubuntu 20.04.5 LTS machine (2 CPU cores, 2.40 GHz clock, 16 GB RAM), different from the one in which the DET is located.
3. **Processing modules**: These modules are the linkers and enrichers presented in Section 3.2, and they are the focus of this work. They process and enhance data prioritising smart city advancement and citizen impact, after which they reinject the newly enhanced data into the Scorpio Broker. Each of the four components is deployed in its separate Docker container, all of them within an Ubuntu 20.04.5 LTS machine (2 CPU cores, 2.40 GHz clock, 16 GB RAM). Their source code is publicly available at [47].

We have gathered data from several heterogeneous data sources in order to refine the processing modules; however, the most relevant example for the scope of this work is SmartSantander [18], which is an IoT infrastructure with several thousands of sensors in the city of Santander, Spain. The data collected from SmartSantander include temperature, air quality, and humidity measurements, among others, allowing a wide spectrum of phenomena to be processed by our modules.

Regarding communication among components, we have used the NGSI-LD API [4], and more specifically batch entity operations and create/update operations from the DET to the broker, and subscription/notification operations from the broker to the enhancement modules. The enhancement modules inject the data back into the broker via batch entity operations and create/update operations.

### 4.2. Results

Each of the enhancement modules developed in this work receives an NGSI-LD entity as an input and provides the same NGSI-LD entity as an output, enriched or linked by leveraging the NGSI-LD API [4], in addition to internal or external knowledge sources depending on the module. In the following subsections, we will present a sample entity before and after being processed by each of the modules, which showcases the experimental results that are obtained after our linking and enrichment.

#### 4.2.1. IoT Data Linker

**Figure 6:**
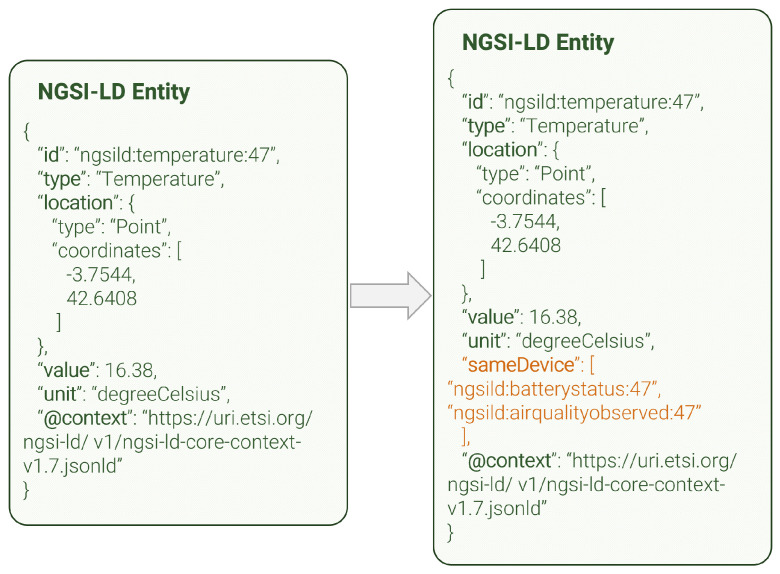
IoT data linker entity enhancement.

The IoT data linker can enhance NGSI-LD entities that originate from an IoT device. IoT devices are typically a collection of multiple sensors (e.g., temperature, humidity, or luminosity). Each of these sensors generates its own separate IoT measurement or observation, which can potentially be hard to reintegrate after data processing due to their only commonality being their device of origin. Figure 6 shows a complete example of a temperature entity before and after being processed by this module. As can be seen, the IoT data linker appends a new NGSI-LD relationship to the entity (so-called *sameDevice*, highlighted in orange) which links it to those other entities originated from the same physical device. In this way, the power of NGSI-LD relationships, which point directly to the related entity’s *id*, allow the user to make much more efficient queries and permit more intuitive navigation.

#### 4.2.2. Geolocation Data Linker

**Figure 7:**
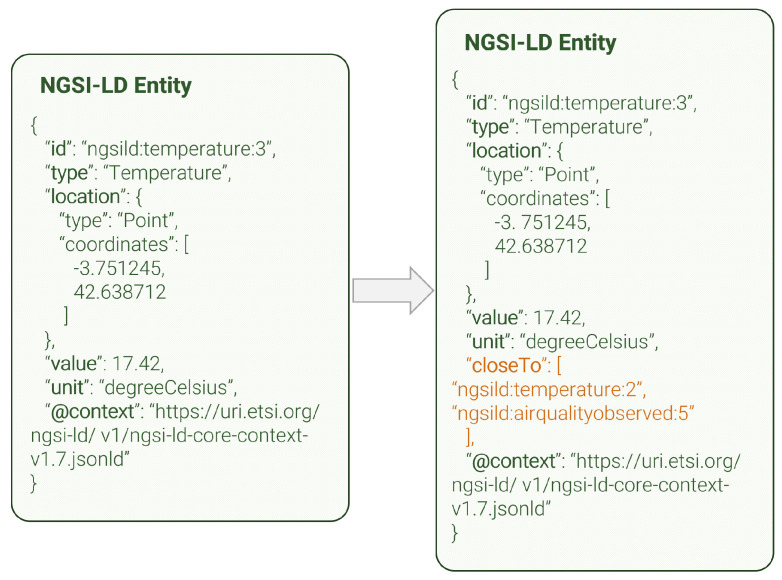
Geolocation data linker entity enhancement.

The geolocation data linker can enhance NGSI-LD entities that have a *location* entitoperty, which is typically the case for most general data and especially smart city-related data, where the exact positioning of measurements is crucial. This module performs a set of geospatial queries to the broker in order to extract those NGSI-LD entities within a certain range of the entity being processed at that moment. In Figure 7, a full example of one such entity is shown, before and after the enhancement. The Geolocation data linker adds a new NGSI-LD relationship (so-called *closeTo*, highlighted in orange) that enables a direct link between NGSI-LD entities within a parametrisable distance of the entity currently being processed. These entities do not necessarily have to be the same *type*, and in fact, this linking process serves as a useful tool to study correlation between several metrics at a given location.

#### 4.2.3. Reverse Geocoding Data Enricher

**Figure 8:**
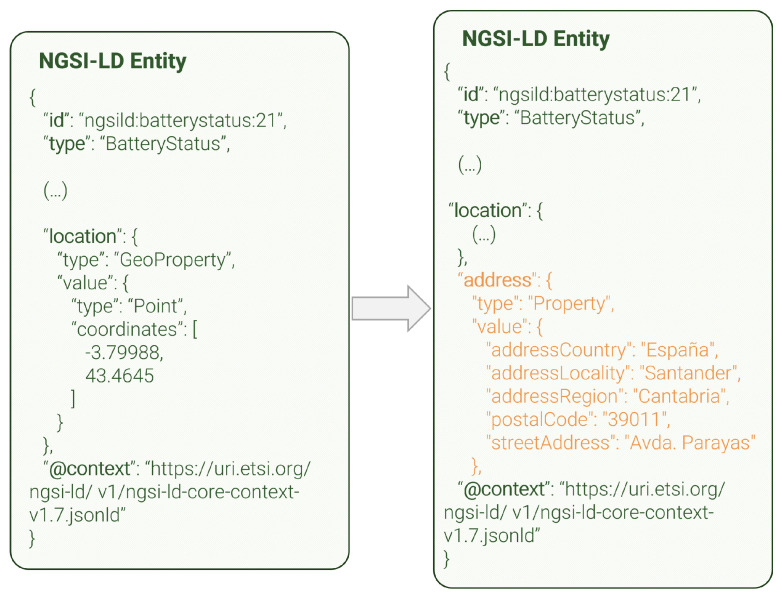
Reverse geocoding data enricher entity enhancement.

The reverse geocoding data enricher, like the geolocation data linker, can enrich NGSI-LD entities that have a *location* entitoperty. It serves as a facilitator for data understandability, as its main purpose is to provide precise information about the location in human-readable form (e.g., street address, postal code…). Figure 8 shows an example of an NGSI-LD entity before and after the enrichment, although not all properties are shown because of length constraints. The reverse geocoding data enricher adds a new *address* property based on the location of the original entity, which is retrieved from a reverse geocoding external service.

It is also worth noting that, unlike the two previous examples, this entity is portrayed in its *normalized* format, i.e., the type of each field (property, geoproperty, relationship…) is shown. We have decided to use the *normalized* formatting in this example, because that way all the details can be appreciated. In the previous examples, these details were not so critical to understand the functionality of the modules, and thus, we chose to use *keyValues* formatting, which is more concise.

#### 4.2.4. Insight Data Enricher

**Figure 9:**
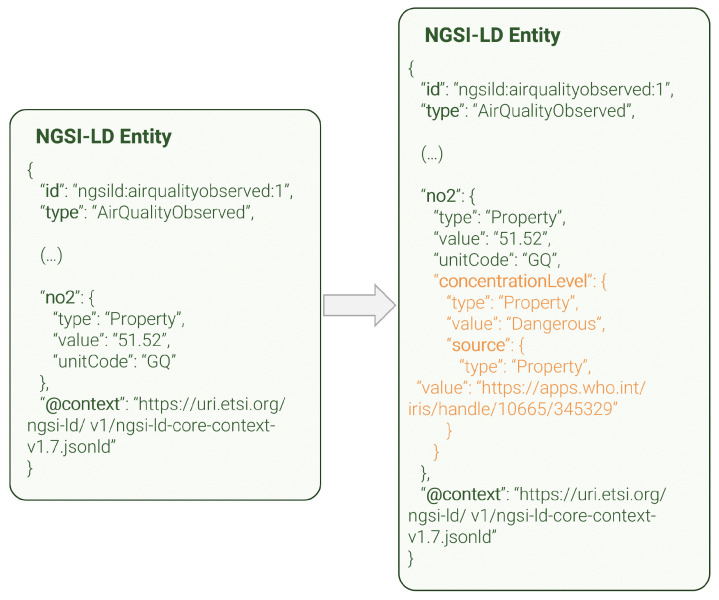
Insight data enricher entity enhancement.

The insight data enricher is more domain-focused when compared with the previous enhancement modules; it can enrich NGSI-LD entities that contain numerical properties within a pre-defined list. The currently supported smart data model types are *AirQualityObserved*, *SoundPressureLevel*, *Temperature*, and *TrafficFlowObserved*. The module maps the numerical value of relevant properties (e.g., NO2, CO2, O3…) and greatly enhances understandability by adding a string-typed sub-property, as can be seen in Figure 9. This figure shows an example of an *AirQualityObserved* entity before and after enrichment, focusing on a single property (*no2*) for clarity reasons. In this case, the sub-property *concentrationLevel* provides an understandable interpretation of the numerical value, that can be complicated to assess by a non-technical user. This sub-property contains a nested sub-property, so-called *source*, which indicates the guideline used to label certain values as certain strings. Figure 9, as Figure 8 above, also uses *normalized* formatting.

The linkers and enrichers presented in this work shift traditional focus towards a more citizen-centred approach. This is achieved by either facilitating navigation through data, enhancing the amount of information available in every entity, or inserting new properties, sub-properties, or relationships that enhance data understandability. From an optimisation perspective, both data linkers reduce the number of queries required to obtain the same information. Moreover, by using more complex NGSI-LD queries (e.g., query all entities whose property *address* has a *postalCode* equal to “39011”, query all air quality entities whose *no2* property has a dangerous concentration level…), information can be retrieved more efficiently by users and applications. This contribution helps not only smart cities in general through more straightforward and efficient data management, but also allows non-technical users (i.e., citizens) to become more familiar with smart city advancements by understanding data and the effects smart cities can have on society as a whole.

## 5. Conclusions

The data linkers and enrichers that have been presented in this work have been designed to continue developing the smart city paradigm forward while ensuring that citizens are not left behind, as opposed to focusing on technical users only. They leverage the principles of linked-data modelling and semantics in their data processing. With a key baseline consisting of a data enrichment toolchain capable of providing NGSI-LD data from heterogeneous sources and an NGSI-LD context broker, these modules have access to several real-world datasets and data streams that serve to experimentally validate their functionality. In fact, the European Data Portal (EDP) hosts several datasets that have been processed by these modules. They are readily accessible at [48].

During the development and implementation of the modules, several limitations and challenges have arisen. Firstly, the NGSI-LD standard is subject to constant evolution, which has resulted in backward compatibility issues when new versions are released. This is particularly true given that NGSI-LD context brokers may implement the standard slightly differently from the specification, leading to missing features or errors. Secondly, finding suitable data for the validation of the modules is not a trivial task. Fortunately, we were able to integrate our work into the DET. Nonetheless, we acknowledge that this may pose a significant challenge for other implementations, especially if the modules are intended to be domain-agnostic. Finally, it is worth mentioning that large volumes of data, which are typical for IoT deployments, may incur additional considerations when it comes to storage and processing capabilities of the hosting machines.

Overall, the semantic enrichment provided by the modules presented in this paper leverages advanced data processing, while keeping smart city improvement as a parallel objective. The key benefits provided include: (1) increasing the value of existing data by providing them with meaning and understandability; (2) enabling more efficient discovery and navigation through such data; (3) establishing links and relationships between data; and (4) enabling non-technical users to benefit from the information that can be extracted from technical data.

Future research in this area involves further analysis of the smart city landscape in order to identify gaps where more enhancement modules could be developed. Most of the identified areas are highly specialised, where more tailored modules are required. At the same time, some of the existing modules (namely, the insight data enricher) can be expanded to support more smart data models, metrics, and guidelines to incorporate into the knowledge base. Additionally, the search for more data sources and frameworks in which to integrate our modules is fostered by their full compliance with the NGSI-LD standard. This means that any platform or framework based on an NGSI-LD context broker will be automatically compatible with our work. This synergy could increase awareness and impact, leading to a potential breakthrough in the field of NGSI-LD in smart cities.

## Data Availability

Data are contained within the article.

## Abbreviations

The following abbreviations are used in this manuscript:

AI	Artificial intelligence
API	Application programming interface
BAD	Big active data
DET	Data enrichment toolchain
EDP	European Data Portal
HTTP	HyperText Transfer Protocol
IoT	Internet of Things
JSON	JavaScript Object Notation
JWTs	JSON Web Tokens
NGSI-LD	Next Generation Service Interfaces with Linked Data
NLP	Natural language processing
SAPP	Semantic Annotation Platform with Provenance
TLS	Transport Layer Security
